# Listening to Japanese Gardens: An Autoethnographic Study on the Soundscape Action Design Tool

**DOI:** 10.3390/ijerph16234648

**Published:** 2019-11-22

**Authors:** Gunnar Cerwén

**Affiliations:** 1 Department of Work Science, Business Economics and Environmental Psychology, Swedish University of Agricultural Sciences, Slottsvägen 5, 230 53 Alnarp, Sweden; gunnar.cerwen@slu.se; 2 Japanese Society for the Promotion of Science (JSPS) International Research Fellow Programme, School of Cultural and Creative Studies, Aoyama Gakuin University, Shibuya, Tokyo 4-4-25, Japan

**Keywords:** soundscape design, sonic experience, tranquillity, noise, garden therapy, landscape architecture, Japanese gardens, autoethnography, soundscape actions

## Abstract

Landscape architecture and urban design disciplines could benefit from soundscape thinking in order to enhance experiential qualities in their projects, though the available tools are not yet fully developed nor tested. The present research aims to substantiate one of the available tools, Soundscape Actions, and thereby increase the understanding of soundscape design. The study focuses on the Japanese garden tradition, which is known for high preference ratings, tranquil qualities and consideration for sound and other sensory experiences. An autoethnographic approach was used to conduct field studies in 88 gardens in Japan, the majority of which are located in urban areas with potential noise disturbance. The studies are based on observations in situ, supported by video documentation, field recordings and readings of sound pressure levels (SPL). A total of 19 Soundscape Actions are described and discussed in the paper. They are structured around three main categories: localisation of functions, reduction of unwanted sounds and introduction of wanted sounds. The study provides concrete examples of how the tool can be used to enhance tranquil qualities, particularly focusing on small green spaces in dense urban settings, involving the (simultaneous) reduction of unwanted sounds and enhancement of wanted sounds/effects. The autoethnographic approach allowed for the phenomenological perspective to be brought forward, which contributed new insights regarding the design tool. The findings are discussed in relation to health and soundscape research, focusing on multisensory experiences, masking strategies and potentials for implementation and future developments of the design tool.

## 1. Introduction

Japanese gardens are sources of inspiration for gardeners, landscape architects and designers around the world [1]. Originally inspired by gardens in China, the Japanese garden tradition has a long history with many particular styles, including the dry landscaped garden *karesansui*, the stroll garden *kaiyū-shiki-teien* and the tea garden *cha-niwa* [2,3]. It is a diverse tradition held together by its Japanese sense of aesthetics, characterised by asymmetry, symbolism, geomancy, careful detailing and the use of natural materials (c.f. [4,5]).

Japanese gardens tend to receive high preference ratings compared to other types of gardens and landscapes [6,7,8,9], which can partly be explained by their informal character and natural expressions [10]. Rocks, water and vegetation constitute some of the most typical materials, meticulously shaped to represent nature “at its best”. For instance, the shape of pruned trees in Japanese gardens have been used as motivation for the “Savannah theory” [11]. Studies have shown that spending time in a Japanese garden can lead to a reduction in heart rate [12] and an improved mood [13]. Some of the most famous gardens are Zen Buddhist gardens, rich with symbolic meaning and spiritual qualities, and sometimes specifically designed to stimulate meditation [3]. Other gardens encourage physical activity and engage the bodily senses [4,14].

The green and natural landscapes of Japanese gardens have constituted an important part of the urban fabric for several hundred years [15]. In contemporary Japan, the gardens still offer a contrast to the restraints of modern life and the highly densified cities that often surround them. Space is a scarce resource in Japan, and gardens are often designed to appear larger than they actually are [16], which makes them interesting as reference objects for pocket parks and other small green spaces.

Today, 55% of the world’s population lives in urban areas, with an expected increase to almost 70% in 2050 [17]. It is known that urbanisation and urban lifestyles can lead to stress and negative health effects [18]. Problems relating to stress constitute a major challenge for the global community. While a certain amount of stress is a natural part of human life, extended periods of exposure may lead to negative health effects, such as sleeping problems, depression, cardiovascular disease and chronic fatigue [19].

Research has shown that nature and nature-like environments can alleviate [20] and prevent [21] such negative health effects. Accessibility to green spaces is thus a crucial factor to consider in city planning, albeit one that may be difficult to adhere to, especially as densification proceeds. Gardens and other small green spaces with natural components may prove to be increasingly important in the future, as their spatial requirements are more flexible than parks. Yet, problems with noise from neighbouring urban activities constitute a challenge. This is the case in Japan, as well as in many other countries around the world.

The sound environment influences the quality of gardens and other green spaces [22,23] and has repercussions on health, both positive and negative [24,25]. It is pertinent to ensure that planning and design is optimised to take this into account. However, it has been repeatedly argued that sound is a limited concern within architectural disciplines and that, if sound is considered at all, it is typically with reference to noise issues [26,27].

Soundscape research, on the other hand, adopts a comprehensive understanding of the sound environment, including problems as well as positive experiences. It is a broad and interdisciplinary field, focusing on the contextual and subjective experience of sound environments. Initiated in the late 1960s [28,29,30], it has gained increased momentum in recent years [31], not least in urban planning and design projects [31,32,33]. In 2014, soundscape was defined by the International Organization for Standardization (ISO) as an “acoustic environment as perceived or experienced and/or understood by a person or people, in context” [34]. To date, a number of tools and approaches have been developed to aid the design of outdoor environments through use of the soundscape approach [35,36,37,38,39], yet few of these have been tested and validated in situ.

In the present study, one of the available tools called Soundscape Actions [27,36] was applied to study sonic experiences in the Japanese garden tradition. The aim was to substantiate Soundscape Actions as a design tool in landscape architecture and to increase the understanding of the design of tranquil soundscapes. Given their health promoting potential [13] and careful consideration of sonic experiences [4,40,41,42,43,44], Japanese gardens were considered an ideal context to appraise the tool, the idea being that the rich knowledge accumulated in a centuries-old tradition could potentially be used to inform future developments of the tool.

## 2. Materials and Methods

**Figure 1:**
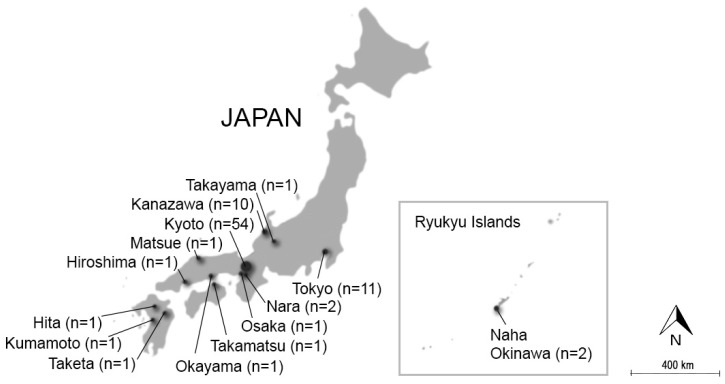
This map illustrates the geographical distribution of the 88 studied Japanese gardens.

The present study is based on empirical material that was collected on site in Japan using an autoethnographic approach [45]. The material was collected during 136 visits to 88 renowned Japanese gardens, the majority of which are located in Kyoto (Figure 1). For further details about the selection of gardens, times of visit and other details, see Appendix A and Section 2.2.

Most of the studied gardens have existed for several hundred years, during which time the gardens and their soundscapes may have undergone change to various extents (not least the soundscapes surrounding the gardens). Alas, it should be pointed out that the study does not aim to contribute with historical insights as to how gardens were designed in different periods or regions in Japan. Rather, the tradition is considered as a context from which to draw general understandings regarding soundscape design which can then be applied to other (contemporary) gardens and green areas. It may well be that some of the effects that were experienced in the gardens were not the result of intentional acts by their designer. However, this does not make it any less interesting for the purposes of the study.

Field notes from the gardens were collected in a digital document amounting to a total of about 23,000 words, which served as the main research material. The design tool Soundscape Actions was subsequently applied to analyse and structure the material. The findings are supported by extracts from the field notes as well as photographs, videos, field recordings and sound pressure level (SPL) readings taken in the gardens. For an overview of video and sound material referred to in this paper, see Supplementary Material—List of video files. For further information on data collection, see Section 2.2.

### 2.1. Using Autoethnography to Substantiate Soundscape Actions

Autoethnography is a qualitative method focusing on the researcher’s own experience and reflection of a phenomenon [45]. It has been increasingly applied in architectural disciplines, where it can be used to uncover various aspects of the cultural practice of designing and experiencing landscapes [46,47,48,49]. In this study, the autoethnographer has a background as a soundscape researcher and a landscape architect. It is in the intersection of these two fields that sonic experiences in Japanese gardens are notated.

Soundscape research has tended to focus on participants’ generalised experiences (e.g., [50,51,52]), while relatively little attention has been paid to how individual subjects experience a phenomenon in detail. The subjective perspective is emphasised in the ISO definition of soundscape [34] and could potentially lead to a better understanding of how contextual factors influence the experience of sound. Subjectivity could also be an important factor when connecting soundscape research to design disciplines; it has been pointed out that architects often use their own subjective site experiences as a starting point when proposing new design solutions [46]. Autoethnography can be used to bring such experiences to the foreground, allowing for comparisons between practitioners [45]. If experiences are shared as autoethnographic narratives, these may also be studied by other researchers and designers as components of their reference libraries. In the present study autoethnography was used to evaluate a design tool, with the intention of substantiating its usage and making it more accessible to practitioners (c.f. [53]).

The approach is akin to the notion of “skilled listeners” as proposed by Hedfors and Berg [54] which has previously been used to study the relationship between sonic phenomena and landscape architecture [55]. In that study, data from skilled listeners were used to provide intricate understandings regarding the experiential qualities of environmental sounds, which were then formulated as a terminology for practitioners. A related approach has also been used by Amphoux [56], involving trained listeners to map urban soundscapes.

The present study differs from the above by focusing on a single listener’s experience, rather than several. As an autoethnographic study it is subjective in nature, and care should be taken when interpreting the results. To ensure validity, efforts have been taken to make subjectivity explicit where it is present [57]. In order to increase generalisability, sonic experiences are discussed in relation to previous research and supported by data collected on site (Section 2.2). In accordance with what has been termed “analytical autoethnography” [58], the findings are used to gain insights on a theoretical framework (Soundscape Actions).

### 2.2. Data Collection

Most of the research was undertaken during 2018, preceded by some initial surveys in 2015. Following the autoethnographic approach, criteria for site selection were guided by a general intention to learn from the Japanese garden tradition in terms of how soundscapes could be designed (see Section 2.2.1 and Section 2.2.2). In total, 88 gardens in multiple locations around Japan were visited (see Figure 1 and Appendix A). The majority of these (n = 54) were located in Kyoto, which is known for its many gardens of high quality. During the spring and autumn of 2018, a total of three months were devoted to field studies in Kyoto. Additionally, a number of gardens in Tokyo (n = 11) and Kanazawa (n = 10) were included in the study, as well as chosen gardens in other locations. The data collection process can roughly be divided into five phases as discussed below. However, it should be noted that the chronology sometimes overlapped. For instance, when a new city was visited, Phases II and III were repeated.

#### 2.2.1. Phase I: Surveying the Field

In the first phase, a survey of previous literature pertaining to Japanese gardens (e.g., [2,3,4,5,14,16]) and their soundscapes (e.g., [40,41,42,43,44]) was undertaken. Contact was also established with key researchers in Japan, leading to some valuable recommendations for gardens to study. Based on the literature review and contact with researchers, prospective gardens were continuously plotted using Google Maps.

#### 2.2.2. Phase II: Initial Study Visits in Gardens

This phase was intended to provide an overview of a wide variety of traditional gardens in Japan. Visits in this phase were relatively short (typically 15–45 min), the intention being to identify gardens that would be able to contribute with insights on soundscape design. Each garden was briefly described in a notebook, including the general impression, the context for the visit, notable features, the date, weather and soundscapes. The notes were brief, though could be elaborated if there were particular sonic features. The notes were taken either directly on site or written down later in the evening of the same day. Each garden was photographed, and in case there were notable sonic features, these were recorded.

#### 2.2.3. Phase III: Extended Visits to Gardens

Gardens that had been noted for further investigation in Phase II were revisited once or several times, with the intention to study selected sonic events more thoroughly. Visits in Phase III were longer, typically lasting more than one hour. The extended time allowed for reflective on-site writing and the capturing of video, SPL readings and field recordings (for technical details, see Table 1). In a few gardens that were difficult to access, Phases II and III were combined.

#### 2.2.4. Phase IV: Summary of Research Material

Garden visits were continuously noted in a Microsoft Excel spreadsheet (Appendix A). Field notes were collected in a digital document, amounting to a total of 23,000 words. This document served as the main research material. Images, video and sound were organised as a digital library that was used as a reference during the process of analysis. Extracts from this library were used to support the findings in this paper.

#### 2.2.5. Phase V: Control

This phase was carried out in conjunction with data analysis, and entailed revisits to some of the previously studied gardens. The intention for these visits was either to confirm findings, make further comparisons between gardens and/or to collect audio-visual material and SPL readings. Most of the work in Phase V was completed in the autumn of 2018.

### 2.3. Analysis of Data

**Figure 2:**
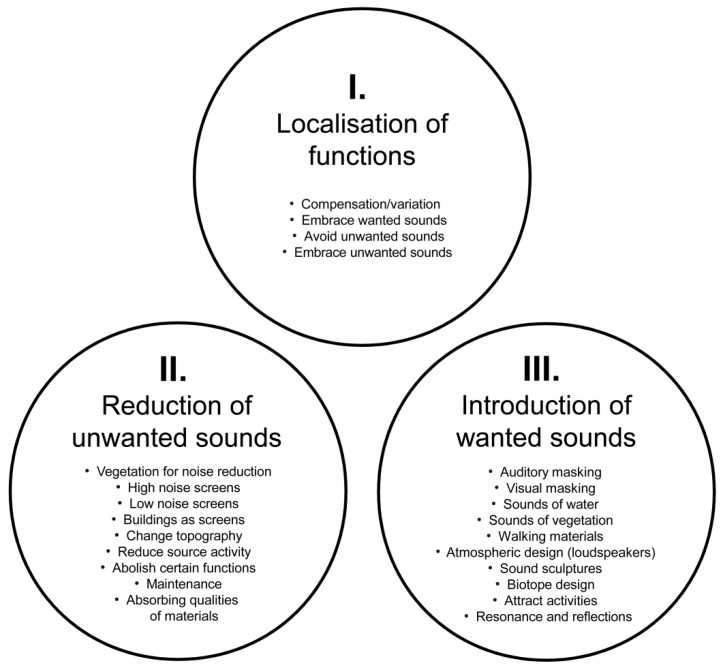
The design tool Soundscape Actions was used as the basis for analysis. In total there are 23 Soundscape Actions divided into three main categories: localisation of functions, reduction of unwanted sounds and introduction of wanted sounds.

The Soundscape Action tool was used as a framework to analyse the research material and present the results. The tool was originally developed in collaboration with practitioners aiming to improve urban areas exposed to noise [27,36,61]. The tool has been described as ”a group of acts that can be taken with the intention of designing a soundscape” ([36], p. 509). There are currently 23 Soundscape Actions divided into three main categories: localisation of functions, reduction of unwanted sounds and introduction of wanted sounds (Figure 2).

The field studies were conducted in 88 gardens around Japan, but the analysis and presentation focuses on gardens located in Kyoto (n = 54). The focus on Kyoto is partly a result of the extensive material collected in this area, and it was also deemed necessary for reasons of clarity. The focus on Kyoto is most evident in Category I (localisation of functions), where other gardens are excluded entirely, so as to allow for comparisons within the city.

Before analysis was initiated, all field notes had been studied and checked for connections to supportive data. In cases where additional data or controls were required, this was noted and carried out (Section 2.2.5). In the analysis, each of the Soundscape Actions was compared with the field notes in order to identify relevant sections in the material; this process was guided by the author’s recollection of the visits as well as strategic searches within the notes. Identified passages were copied to a new Microsoft Word document which was used as a base from which to summarise the findings. Supportive data was compared with the Soundscape Actions and, where applicable, links were provided in the new document.

It was found that four of the 23 Soundscape Actions were not applicable to the given context—embrace unwanted sounds, abolish certain functions, low noise screens and atmospheric design (loudspeaker-based)—and have thus been omitted from Section 3, leaving a total of 19 Soundscape Actions. These four are further described and discussed in Section 4.

## 3. Soundscape Actions in Japanese Gardens: Descriptions and Findings

The results are structured according to the three main categories illustrated in Figure 2. Each of these three categories is first given a brief *Category introduction* including any particular *Prerequisites and delimitations* of importance to the study. The 19 subcategories (Soundscape Actions) are then introduced using the following three headings: *Description*—A brief definition of each Soundscape Action based on previous studies; *Gardens of particular interest*—A list of gardens that were important for the findings presented and; *Findings*—A description of how the Soundscape Action related to the studied Japanese gardens and the findings that were made. Where applicable, connections to previous research are indicated.

### 3.1. Localisation of Functions

*Category introduction*: The first main category in the Soundscape Action tool regards the localisation of functions in relation to their surroundings (in the present study: gardens in relation to their urban surroundings). This category typically entails the consideration of noise and the compatibility between functions.

**Figure 3:**
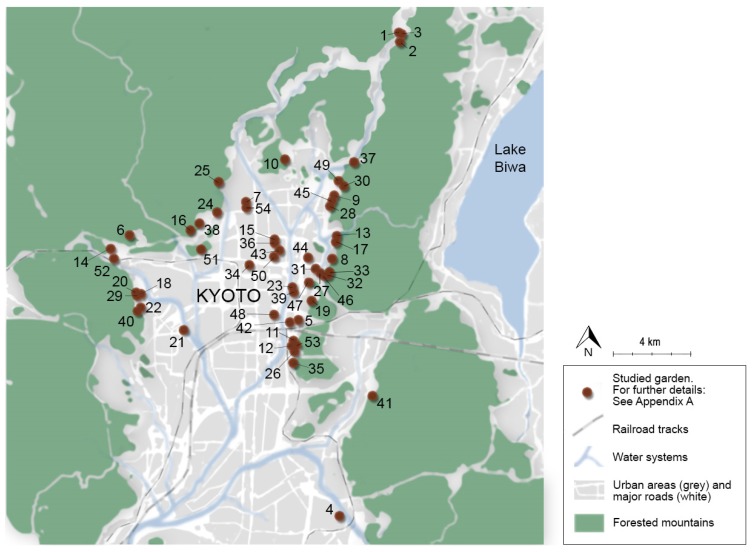
This map illustrates the locations of the 54 gardens that were studied in and around Kyoto. See Appendix A for names and further details of the study visits.

*Prerequisites and delimitations*: The Soundscape Actions in this category are mainly described based on observations and comparisons between gardens, complemented with the mapping of garden locations, images and SPL readings. For the purposes of clarity, all gardens mentioned in this main category are limited to one city, i.e., Kyoto (Figure 3). While acknowledging the considerable age of many gardens and the complex development of the city over time, the study focuses on how the gardens and their contexts are conceived today.

#### 3.1.1. Compensation/Variation

*Description:* Compensation/Variation acknowledges the differences between neighbouring soundscapes as a potential quality. Gardens are frequently referred to as “safe havens” and “oases”, thus implying a relative tranquillity and quietness where it is possible to escape from the hustle of the outside (urban) world. Consequently, busy urban settings surrounding a garden may be regarded as an asset, by offering contrast and juxtaposition (provided that the tranquillity inside the garden can be maintained).

*Gardens of particular interest:* Ōhashi-ke (35), Konchi-in (27), Murin-an (31).

**Figure 4:**
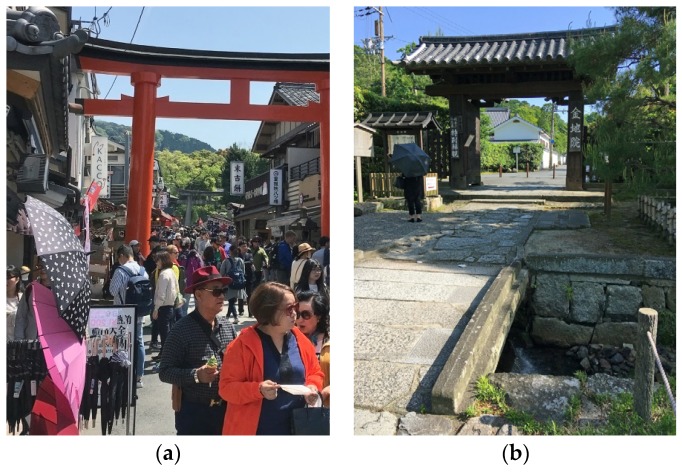
Intense soundscapes may work as a contrast to enhance tranquillity in nearby gardens. (**a**) A busy tourist street at Fushimi Inari-Taisha in Kyoto. (**b**) The entrance to Konchi-in temple leads the visitor across a loud water stream.

*Findings:* The notion of Compensation/Variation was particularly pronounced in lesser known gardens located close to busy tourist routes. For instance, Ōhashi-ke garden in southeast Kyoto is located only two to three hundred metres from the immensely popular Shinto shrine Fushimi-Inari Taisha. In this intense tourist area, the narrow roads are filled with visitors en route to or returning from the shrine, lingering among market stalls and food shops (Figure 4a). In the study, the overwhelming intensity around Fushimi-Inari Taisha was found to influence the experience inside Ōhashi-ke by making the garden seem calm and quiet in comparison. Hence, the experience of the garden’s soundscape depended not only on sounds experienced inside the garden, but also on previous encounters in the surrounding area. This polarity was captured in a comparative recording between the two sites (Video S1, https://vimeo.com/350108144).

A similar effect of contrast can be experienced about 5 km north, in Konchi-in garden, where the effect is articulated through the sound of water (in addition to the proximity of a tourist route). In order to reach the garden, the visitor needs to cross a loud water stream (Figure 4b). On the bridge across the water, the SPL temporarily reaches over 60 decibels (62 dBA; 21 April 2018), but drops rapidly as the main gate is approached on the other side. Inside the garden itself, the SPL is about 20 decibels lower (38–43 dBA, as observed in different parts of the garden; 21 April 2018). This example illustrates how the addition of sound could be intentionally used to temporarily raise the SPL in order to produce a relative tranquillity at the offset. The effect could arguably be further enhanced if it were combined with screens to increase the contrast. Such a combination can be experienced in another garden in the vicinity, Murin-an, though only occasionally, as the channel that produces the sound is dependent on rainfall to reach sufficient water flow.

#### 3.1.2. Embrace Wanted Sounds

*Description:* To embrace wanted sounds is to make use of pre-existing sounds as a quality when selecting the locations of new functions. For instance, by selecting a location next to a forest, this may add the sound of twittering birds and rustling vegetation, among other experiences.

*Gardens of particular interest:* Nanzen-in (32), Sanbō-in (41), Entsū-ji (10), Shisen-dō (45), Sanzen-in (2), Ruriko-in (37).

*Findings:* Kyoto is surrounded by wooded mountain ranges on three sides: Higashiyama to the east, Kitayama to the north and Nishiyama to the west. A substantial amount of the most well-known gardens in Kyoto are located on the fringes of the city (c.f. Figure 3), often bordering the foothills of the mountain ranges directly. Not only is this a scenic setting which offers good views, but the proximity to nature also creates good prerequisites for a rich soundscape, inviting the sound of rustling leaves, purling water streams and the twittering of birds to be “borrowed” from the surrounding mountains:

Nanzen-in is an exquisitely designed small pond garden, parts of which date back to the 13th century. Lush mountainous woodlands surround the garden and add to the atmosphere through fragrance and sound. In the southeastern corner of the garden, a waterfall supplies fresh mountain water to the ponds.

**Figure 5:**
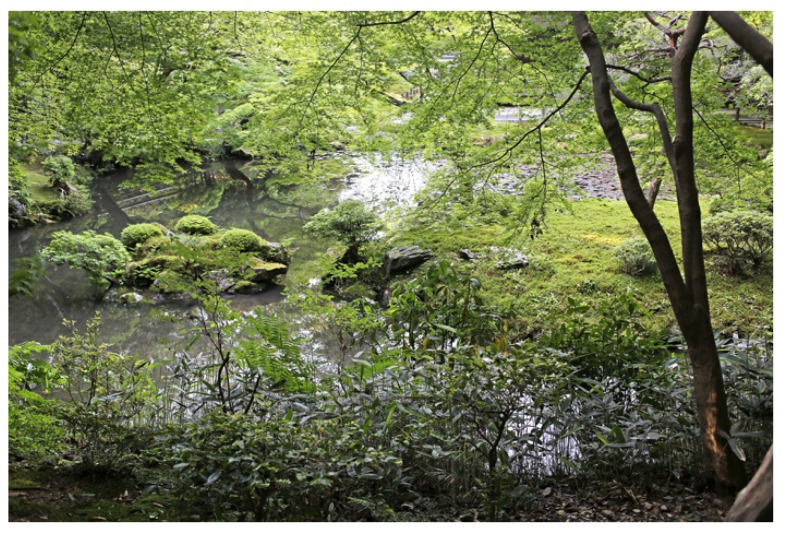
Nanzen-in, a sub-temple to the Nanzen-ji complex, has a small pond garden that borders mountainous woodlands attractive to birds and other animals.

Nanzen-in garden; 14 May 2018 (Figure 5)

In the Japanese garden discourse, *shakkei* is an important concept used to describe a technique whereby scenic features from the surrounding landscapes are “borrowed” and incorporated as part of the garden design. A typical example is when a distant mountain is framed to become part of the garden, articulated by carefully pruned vegetation. The notion of *shakkei* has mostly been discussed as a visual concept [62], however it is also fruitful to consider it as referring to sound and the other senses (c.f. [42]). This is exemplified in Nanzen-in above, as well as in other gardens like Sanbō-in (Video S2, https://vimeo.com/312665806), Entsū-ji, Shisen-dō, Sanzen-in and Ruriko-in.

#### 3.1.3. Avoid Unwanted Sounds

*Description:* Avoiding unwanted sound is about considering existing sound sources that could potentially be disturbing and to make sure to locate new functions in areas shielded from such sources by providing sufficient distance and/or using shelter from buildings, mounds and other existing features.

*Gardens of particular interest:* Enkō-ji (9), Rurikoin (37), Shisen-dō (45), Konpuku-ji (28), Giou-ji (14), Saihoji (40), Shōsei-en (48).

*Findings:* Studying several gardens in the same city made it possible to make comparisons between different locations. While the majority of studied gardens were located on the fringes of the city, others had more urban locations, and thus provided an important reference. Almost all gardens located on the fringes of the city were perceived as quiet. Enkō-ji is an example of one such garden, where the background ambience was found to be around 40 dBA (12 May 2018). In that example, most of the ambience consisted of subtle natural sounds from the garden and the surrounding forest. It was striking how the low SPL made it possible to hear “further away”:

I measure the background ambience at the veranda to be around 40 dBA. The ambience consists of a stream, distant birds and the occasional wind in the trees. In a way, it is so quiet that it seems I can hear further away.

**Figure 6:**
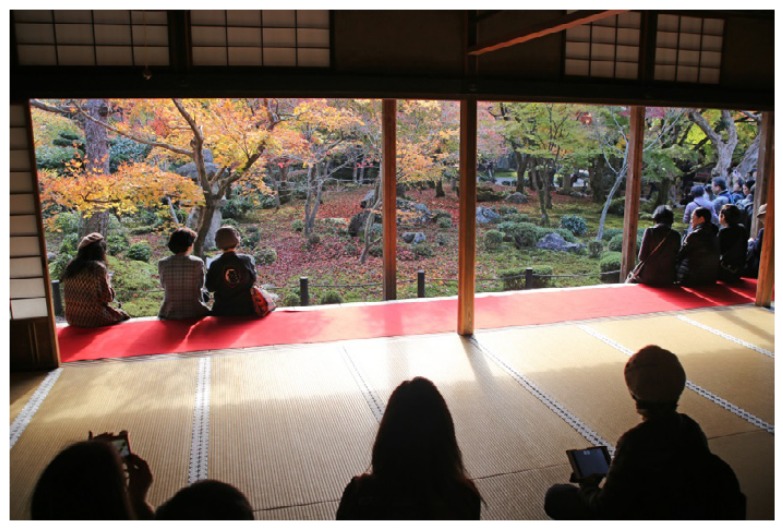
Autumn in Enkō-ji temple. The sliding doors to the main hall have been opened, thus allowing the sounds and sights from the garden to enter into the building.

Enkō-ji; 12 May 2018 (Figure 6)

Needless to say, gardens in central locations tended to be more exposed to noise, inevitably leading to reduced dynamics and a shorter “acoustic horizon” [30]. Gardens in urban locations could still be experienced as surprisingly tranquil, partly owing to the extensive use of water streams with masking capabilities (see further in Section 3.3.1. Auditory Masking). Other important factors for reducing noise include distance to major roads, use of vegetation and screens (garden walls and other structures).

The most problematic of the gardens studied in Kyoto was Shōsei-en, located close to the central station. Shōsei-en is a large garden, and the disturbances varied quite extensively inside the garden (45–60 dBA, as observed on different locations in the central parts of the garden; 29 April 2018). Such local variations constitute important prerequisites when functions within a garden are planned, as some features are more sensitive to noise than others.

### 3.2. Reduction of Unwanted Sounds

*Category introduction:* Soundscape Actions in this main category concern the reduction of unwanted sounds, most typically sounds from urban settlements and related activities like traffic. In contrast to the previous category, these Soundscape Actions are performed after the relative locations of functions have been decided upon.

*Prerequisites and delimitations:* Descriptions in this category are mainly based on observations. It was not deemed fruitful to use SPL to assess noise reduction in most cases, the reason being that this would have required comparisons that could not be obtained due to practical reasons (such as observing SPL with and without a garden wall). Instead, the studies focused on how various features in the gardens were designed, and discussed this in relation to previous research. The focus remains on gardens in Kyoto, though other examples are included as well.

#### 3.2.1. Vegetation for Noise Reduction

*Description:* Vegetation is commonly used to reduce the impact of noise; one of the most typical applications is vegetation belts along roads. However, the actual effect of vegetation for reducing SPL is debated [63], most likely because the effect varies extensively depending on a number of factors such as vegetation type/s, distribution pattern, and the absorbing qualities of the ground cover [63,64,65]. Psychological factors are also important, in particular source visibility [66] (see further in Section 3.3.2. Visual Masking). Moreover, the sound of vegetation can have a positive masking effect that shifts focus from noise (see also Section 3.3.1. Auditory Masking and Section 3.3.4. Sounds of Vegetation).

*Gardens of particular interest:* Saihoji (40), Murin-an (31), Shin’en (44), Chishaku-in (5).

**Figure 7:**
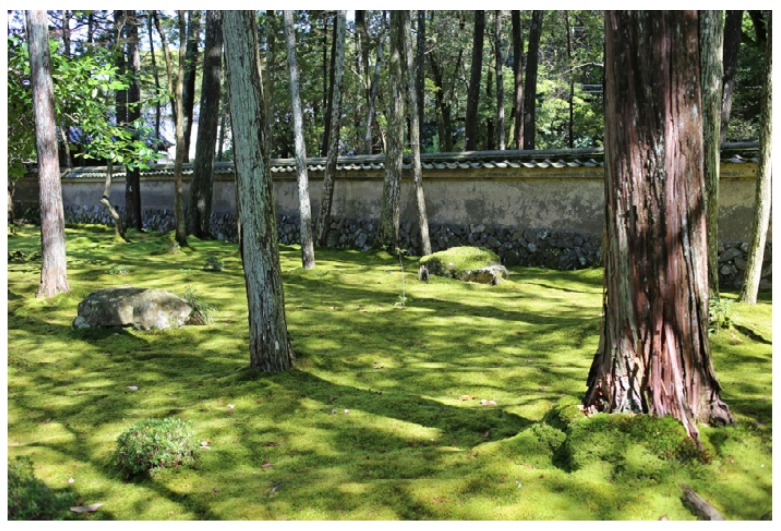
Moss and a garden wall in the woodland of Saihoji in Kyoto.

*Findings:* Woodlands constitute a recurring feature in Japanese gardens, often found together with moss as in the famous Saihoji: the moss temple (Figure 7). Woodlands’ effect on noise reduction is multifaceted. The trees themselves have some effect, depending on species composition, stem thickness, stand density and other factors [63,64,65], though there are several indirect factors that need to be taken into consideration, such as interference from wind. Wind is known to carry noise, hence woodlands can limit noise propagation in some situations [64]. For gardens, the wind reduction is particularly important along garden walls where there is otherwise a risk of micro-metrological turbulence that can have extensive negative effects.

Almost all Japanese gardens in the study had garden walls, and these were often combined with vegetation. The combined effect of vegetation and wall is beneficial, not only for its enhanced noise reduction [64], but also as a means of articulating the garden space and delimiting it from the outside world (Video S3, https://vimeo.com/311434655, see further in Section 3.3.2. Visual Masking). Moreover, wind causes trees to rustle and this sound (together with birds and sounds from other animals attracted to the forest) can have the effect of masking noise (see further in Section 3.3.1. Auditory Masking and Section 3.3.7. Biotope Design).

Another aspect of woodlands is the effect that trees have on the ground cover. For instance, moss is one of the most characteristic features in Japanese gardens and it seems to thrive particularly well in moist woodlands, where it is shaded by the tree canopy (see further in Section 3.2.7. Absorbing Qualities of Materials). Moss is interesting for present purposes as it has a good ability to absorb ambient noise, an ability it shares with organic soil, grass and other soft ground covers (c.f. [67]).

#### 3.2.2. High noise Screens

*Description:* A screen or wall with the approximate height of 1.8 metres or above is a high noise screen. Like all screens, they should be located as close to the source as possible [68]. High noise screens make it difficult for most people to see to the other side of the screen (unless the screen is transparent).

*Gardens of particular interest:* Murin-an (31), Shin’en (44), Saihoji (40).

*Findings:* Garden walls constitute a characteristic feature in the Japanese garden. Traditionally, the walls are made from clay, resting on a foundation of rocks and with a tile roofing (Figure 7), yet there are also examples of constructions based on other materials such as wood and stone. The height of garden walls vary; most are tall enough to offer visual seclusion, and some are three metres or higher, thus suggesting that screening of noise might have been an important consideration in addition to visual/spatial seclusion. Walls can be used to define the edges of a garden as well as having an effect on noise reduction from the outside. The actual noise reducing effect varies depending on several factors, and the reduction is most effective when the screen is located close to the source (or alternatively close to the receiver) [68]. As previously discussed, vegetation can improve the effect [64].

#### 3.2.3. Buildings as Screens

*Description:* This Soundscape Action acknowledges the potential of strategically located buildings to reduce unwanted sound. Buildings can be effective noise screens, especially if they are tall and made of solid material [38]. They may also be combined with traditional noise screens.

*Gardens of particular interest:* Enkō-ji (9), Shisen-dō (45), Rurikoin (37).

*Findings*: Most buildings in and around the studied gardens were built in traditional style. One characteristic feature of traditional Japanese buildings is that they are made of light materials. In fact, some parts are even made of paper, including the *Shōji*, sliding doors. Moreover, the buildings are regularly opened up to the garden (c.f. Figure 6). In this respect, traditional buildings are not ideal when it comes to noise reduction, and the lower frequencies particularly are able to pass through buildings easily. However, these limitations with traditional buildings were seldom experienced as problematic in the study. One reason could be that the buildings tended to be located inside the boundaries of the garden walls and/or that they were located with sufficient distance from the noise.

There is an opposite and positive effect associated with the traditional buildings’ ability to carry sound. The light construction can be said to “invite” sounds from the garden such that these may be experienced from inside the building, making it possible to listen to water, birds, the chanting of monks, the occasional bell, the murmur of other visitors or any other sound in the vicinity. Japanese buildings are known for their ability to blur the borders between inside and outside [14], and sound plays an important part in this.

#### 3.2.4. Change Topography

*Description:* Land masses can be shaped to form strategic topographical patterns like mounds and/or valleys, which can be used to screen noise. The effect varies depending on factors such as height, width, detailed shape as well as planted vegetation (if any) [64].

*Gardens of particular interest:* Katsura Rikyū (21), Kōrakuen (76), Koishikawa-Kōrakuen (59) Suizen-ji Joju-en (77), Rikugi-en (64), Adachi Museum garden (79), Goten (Ninna-ji) (16).

*Findings:* Topographical features constitute a recurring design feature in the Japanese garden. In Katsura Rikyū in Kyoto, for instance, a pond was dug out and the soil was used to create an artificial mound, *tsukiyama* [4], where a tea house is located. Topographical features offer good views over the landscape, but they can also be visually striking in their own right, such as the characteristically shaped hills in Kōrakuen in Okayama and Koishikawa-Kōrakuen in Tokyo. Mounds are often designed to represent famous and/or sacred mountains, in which case they have a symbolic value [2,3]. The almost perfectly shaped grass cone in Suizen-ji Joju-en, Kumamoto, made to look like Mount Fuji, is a good example.

Topographical features constitute a useful means to reduce noise, if strategically located [64]. However, neither of the features mentioned above seem to have been built with the deliberate purpose of screening noise from the outside, as they are typically located in the central parts of the gardens where the effect should be limited, given the distance to the source [68].

On the other hand, it has been suggested that topographical features in some Japanese gardens are used with the intention of reducing the sonic impact of waterfalls inside the gardens [4]. The idea is that by “muffling” the sound of a waterfall in this way, it may seem to be further away than it actually is, hence making the garden appear larger (c.f. *shakkei* in Section 3.1.2). This effect was encountered in three gardens: Rikugi-en in Tokyo (Video S4, https://vimeo.com/312519101), Adachi Museum garden outside Matsue and Goten (Ninna-ji) in Kyoto.

#### 3.2.5. Reduce Source Activity

*Description:* This Soundscape Action is concerned with everyday activities in the environment. It focuses on how unwanted sound from such activities can be reduced by decisions made in planning and design, such as enforcement of speed limits on roads or restrictions on social behaviour.

*Gardens of particular interest:* Tenryū-ji (52), Daisen-in (7), Shisen-dō (45), Goten (16).

**Figure 8:**
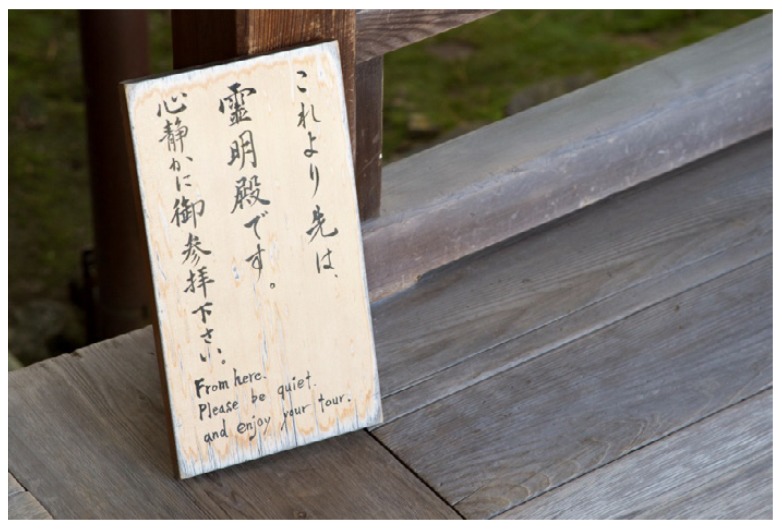
A sign in Goten, Kyoto, encouraging people to be quiet during their visit: “From here. Please be quiet and enjoy your tour.”.

*Findings:* Many Japanese gardens are popular attractions, and it is common to experience sounds from other visitors. These sounds can be quite intense, particularly in famous gardens with high accessibility, like the UNESCO World Heritage Site Tenryū-ji, which attracts many tourists. Taking photographs is a popular activity inside the gardens, and one which generates a lot of sound including discussions, shutter sounds and so on. Some gardens, like Daisen-in, do not allow photography, and this seems to have a positive effect on the soundscape as well as corresponding better with the “mindful” spirit of zen in that temple. In some gardens, like Shisen-dō and Goten, there are signs encouraging people to be respectful in their visits (Figure 8).

#### 3.2.6. Maintenance

*Description:* Maintenance can generate a lot of noise, particularly when combustion engines are used. On the other hand, the use of hand-driven equipment and/or animals can potentially add a quality to the soundscape. Maintenance can be controlled in maintenance plans, but it is also influenced by design solutions. For instance, a lawn requires constant mowing, while a free-growing meadow is less intensive.

*Gardens of particular interest:* Ginkaku-ji (13), Rurikoin (37), Konchi-in (27), Shūgaku-in Rikyū (49).

**Figure 9:**
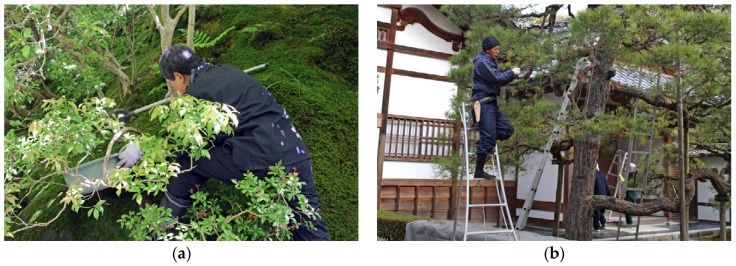
(**a**) A garden worker sweeping the moss in Rurikoin, Kyoto. (**b**) A garden worker pruning a pine tree in Ginkaku-ji, Kyoto.

*Findings:* Generally speaking, Japanese gardens are intensive when it comes to maintenance. Fallen leaves and other litter are removed meticulously, as are weeds. Moreover, the characteristic shape of trees and shrubs requires extensive pruning to be maintained and the gravelled areas of the dry gardens, *karesansui*, are raked every morning. On the other hand, the widespread use of moss rather than grass reduces the need for lawn mowing. Despite the intensive care required, as well as the fact that a lot of maintenance is carried out during opening hours, the work was rarely perceived as disturbing during the study. To a great extent, this might be ascribed to the fact that most tasks are performed by hand. The soft clipping of secateurs or the sweeping of a broom are sounds that add a layer to the experience, rather than disturb it (Figure 9a,b and Video S2, https://vimeo.com/312665806).

Modern equipment is unusual, but was noted on a few occasions when it became quite disturbing. For example, in a garden normally perceived as a quiet gem, Konchi-in, a leaf blower was encountered on one visit, raising the SPL from 41 dBA to 65 dBA (15–20 m from the machine, 14 May 2018). Leaf blowers were also noted on two visits to Shūgaku-in Rikyū, though the garden workers there waited until the visitors had passed before using the machines (visits to Shūgaku-in Rikyū are organised in groups with a guide).

#### 3.2.7. Absorbing Qualities of Materials

*Description:* This Soundscape Action regards the strategic application of materials with absorbing qualities, such as vegetation soil and moss, to reduce noise impact.

*Gardens of particular interest:* Saihoji (40), Rurikoin (37), Enkō-ji (9), Sanzen-in (2).

*Findings:* The prerequisites for moss are good in Japan, as the proximity to the ocean creates a humid climate which helps the moss to thrive. The timeless expression and subtle atmospheric appeal of moss corresponds with the Japanese aesthetics known as *wabi-sabi* [69] and many gardens cultivate moss intentionally. Moss can have a positive effect on the sound environment, as it grows to create a soft and absorbing surface on the ground (c.f. [64,67]). The famous moss temple Saihoji, for instance (Figure 7), was found to be one of the most quiet gardens in the field studies. The ambience at the entrance to the pond section of the garden was noted at 35 dBA (18 November 2018). As speculated during an earlier visit to the garden, moss could be one of several explanations for the quietness:

I attribute the silence partly to the fact that the garden is situated in the outskirts of Kyoto, making it less likely to be affected by city noise. However, the silence can also in part be attributed to the extensive coverage of moss, which is known to absorb sound. Moreover, the garden is surrounded by walls on the one side, and hills on the other, to offer a shelter from the surroundings.

Saihoji; 15 May 2018 (Figure 7)

### 3.3. Introduction of Wanted Sounds

*Category introduction:* Soundscape Actions in this category can be used to introduce, stimulate or enhance sounds that are considered wanted in a particular situation. Needless to say, wanted sounds vary depending on context and individual preferences along with a number of other cues [51,52,70,71], yet sounds from nature, like water, birds and rustling vegetation, are typically considered to be wanted sources [50].

*Prerequisites and delimitations:* Descriptions of Soundscape Actions in this category are based on subjective observations supported by field recordings, images and SPL readings. The focus remains on gardens in Kyoto, but other examples are included as well.

#### 3.3.1. Auditory Masking

*Description:* Auditory masking is an effect where one sound (masker) is used to reduce the impact of another sound (target). Auditory masking has been used in a number of urban design projects, typically involving water features to reduce the impact of traffic noise [72]. Essentially, there are two types of auditory masking: energetic masking and informational masking [73]. In energetic masking the masker sound literally covers the target sound, rendering it inaudible. It has been found that, in the case of urban water features, an increase of about 8–10 dBA is required to achieve this effect [74]. In informational masking on the other hand, both sounds are audible, yet the focus shifts from the target to the masker.

*Gardens of particular interest:* Eikan-dō (8), Murin-an (31), Shōsei-en (48), Koishikawa-Kōrakuen (59), Nezu museum garden (63), Kenroku-en (68).

*Findings:* The ample use of water features in Japanese gardens constitutes an ideal setting to study auditory masking. The notion of energetic masking was conceived to be relatively straightforward. It would typically be experienced in the proximity of loud water features, where all surrounding sounds were effectively covered. Informational masking, on the other hand, was found to be much more complex, involving several cues including the physical characteristics of the inherent sound sources and their relative locations in space. The studies also indicate that informational masking could be affected by visual information (c.f. Section 3.3.2. Visual Masking), as well as the notion of gestalt psychology (c.f. [30,75]), as the following experience from Eikan-dō illustrates:

A loud stream and two waterfalls border the garden on its southern side. In a way, the powerful sound seems to be reinforcing the edge of the garden, which is also marked by a fence. Behind the fence, there is a high school and a kindergarten. This creates an interesting and potentially disturbing situation, as the sound of the children playing can collide with the activities in the temple, activities that presumably are best performed in a tranquil and quiet environment. The sound of children playing is loud, but it is partly masked by the powerful sound of the water. It can still be heard through the water, but in a way, it is as though it becomes located on a different spatial layer, or to use gestalt psychology, in the background. Without the water, the sound of children would probably have been much more intrusive.

**Figure 10:**
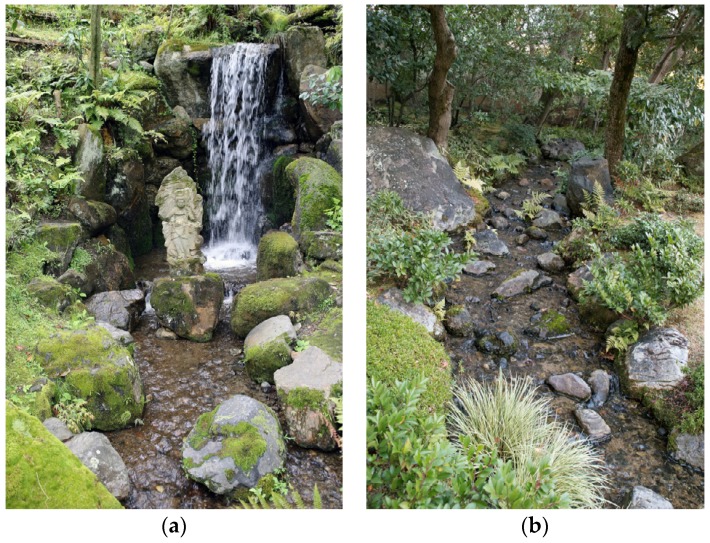
Loud water features can be used to mask outside noise. (**a**) A waterfall in Eikan-dō, Kyoto. (**b**) A stream in Murin-an garden, Kyoto.

Eikan-dō; 18 April 2018 (Figure 10a)

Sound level is an important factor to consider in relation to masking strategies. It has been argued that if the SPL reaches above 65–70 dBA, all sounds start to become disturbing, in which case masking strategies are not fruitful [39]. To achieve tranquil qualities, much lower levels than this are needed. It has been shown that good soundscape quality in suburban green areas and city parks typically require a level below 50 dBA [76] and that informational masking is more efficient at levels below 52.5 dBA (traffic noise) [77]. In Eikan-dō, the SPL was 58 dBA (29 November 2018) at the point where the above description was noted, a few metres from the stream that borders the garden’s edge.

#### 3.3.2. Visual Masking

*Description:* The idea behind visual masking is that if the source of a noise is hidden from view it is less likely to attract attention and cause disturbance. It has been argued that visual masking can be a fruitful strategy as long as the noise that is being hidden is not too prominent [66,78]. This strategy has also been referred to as source (in)visibility [66] and is related to the notion of acousmatics [79].

*Gardens of particular interest:* Murin-an (31), Shin’en (44), Chishaku-in (5), Eikan-dō (8), Shōsei-en (48).

*Findings:* As previously discussed, Japanese gardens make extensive use of garden walls combined with vegetation to mark the edges of the premises. The clearly defined visual spaces keep the visitor’s attention within the garden, presumably making noise from the outside world less likely to be noticed (Video S3, https://vimeo.com/311434655). In a similar manner as auditory masking discussed above, visual masking seems to entail a kind of layering of experiences in terms of background and foreground. In fact, it was found that these two masking effects may work together, as the following example from Murin-an garden in Kyoto illustrates:

It seems the garden wall manages to shield the sound from the street well; the combined effect of screening and lack of visual contact makes me feel as though the cars belong to another world. Moreover, I think it helps that the sound of cars mixes nicely with the rustling of vegetation above the wall, as it is a windy day when I visit. In fact, the vegetation follows the exact same stretch as the road, but because only the vegetation is visible from where I sit, it is easy to partly ascribe the sound of the traffic to the vegetation. Suddenly, a bus passes by, I can clearly see its white roof above the wall, and the illusion is broken for an instance. The sound seems disturbing now, partly I think because I can see the bus, and partly because the sound is louder; I can even feel the vibration from where I am sitting. As the bus passes away, it becomes quiet again.

Main building, Murin-an; 6 May 2018 (Figure 10b)

The ambience in the example (without passing cars) was noted at around 46 dBA (6 May 2018). The rather high wall surrounding the main building shields noise effectively, and the SPL was only raised by a couple of decibels when cars were passing by, thus helping to keep the noise “not prominent” [66]. As a reference, outside the wall on the sidewalk, the ambience was noted at around 54 dBA, which was increased by more than 10 dBA as a car passed by (14 May 2018).

#### 3.3.3. Sounds of Water

*Description:* Water has the potential to create a vast amount of sonic effects, ranging from the powerful broadband noise of a waterfall through to the subtle tickling of a single drop. Water can be made to vary in tone, rhythm and strength [80,81]. It is generally perceived as a pleasant sound, though can be annoying depending on the character [82]. Water features have repeatedly been used to produce masking effects from urban noise in strategic locations [32,72,74].

*Gardens of particular interest:* Tsujike teien (75), Kenroku-en (68), Murin-an (31), Eikan-dō (8), Shin’en (44), Ginkaku-ji (13), Funda-in (12), Rurikoin (37), Chishaku-in (5), Tenryū-ji (52).

**Figure 11:**
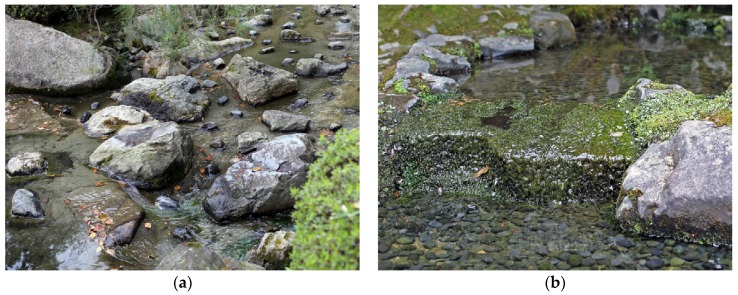
Two strategies to articulate water sound in Japanese gardens. (**a**) Stones are strategically laid out to enhance the sound of a water stream in Shin’en garden (Heian Jingu Shrine), Kyoto (Video S5). (**b**) A barely audible miniature waterfall in Ginkaku-ji, Kyoto (Video S6b).

*Findings:* The ample rainfall and mountainous geography of Japan supplies many gardens with direct access to natural water with a high flow rate. As a general trait, water features in Japanese gardens tend to echo expressions that can be found in nature (Figure 10a,b and Figure 11a,b). For instance, Japanese gardens make frequent use of ponds, natural streams and waterfalls, while formalistic expressions like fountains are less frequent.

Streams and waterfalls with loud and roaring sounds can be found in Tsujike Teien and Kenroku-en in Kanazawa, as well as Murin-an and Eikan-dō in Kyoto. Such loud water features are particularly useful to mask out urban noise through energetic masking (c.f. Section 3.3.1. Auditory Masking). In some gardens, stones are used to deliberately enhance the sound of streams. In Shin’en garden in Kyoto, a stream that connects the eastern and northern ponds is narrowed by the use of boulders, which increases the speed and directs the flow (Figure 11a and Video S5, https://vimeo.com/311170428). As the water descends towards the next level, rocks are strategically located to break the flow. This makes the stream full of life and produces a stronger and more interesting sound than would otherwise have been the case. This technique can be traced back to at least the 11th century in Japan, where it is mentioned in the classic novel *The Tale of Genji*: “The stream above the waterfall was cleared out and deepened to a considerable distance; and that the noise of the cascade might carry further, he set great boulders in mid-stream, against which the current crashed and broke” ([83], p. 428).

If some water features are created in a way that enhances their sound, it is also common to find opposite and more delicate approaches where extremely low sound levels are emphasised. In some cases, such kinds of subtle water features are in fact just above the audibility threshold. They are often found in zen gardens, which could suggest that they are used to support meditation practices (c.f. [3]). The expressions vary from the tranquil dripping of water in a small washing basin, *chozubachi,* as experienced in Funda-in in Kyoto, through to what could essentially be described as miniature waterfalls, found in gardens like Rurikoin, Chishaku-in (Video S6a, https://vimeo.com/311182675) and Ginkaku-ji (Figure 11b and Video S6b, https://vimeo.com/311079936) in Kyoto. Another expression of this kind of aesthetics can be found in the “dry waterfall”, *karetaki*, (e.g., Tenryū-ji in Kyoto), where stones are arranged to look like a waterfall but intentionally kept dry, thus only suggesting the sound [43].

#### 3.3.4. Sounds of Vegetation

*Description:* Vegetation sound is perhaps most commonly associated with leaves that rustle in the wind. The character of the rustle varies depending on several factors, including species composition, spatial layout and wind [27]. Tree branches can produce crackling sounds if they are swaying in the wind, and large leaves can enhance the sound of rain as raindrops fall on them [84]. Some species are known as good rustlers, including the poplar genus (aspen) and bamboo [84,85].

*Gardens of particular interest:* Funda-in (12), Chishaku-in (5), Rikugi-en (64), Rentaroh Taki’s Memorial garden (81).

*Findings:* The sound of rustling leaves could be experienced in most gardens, the character varying from a tranquil and subtle atmosphere to strong gusts. On windy days, when the effect was most pronounced, the leaves could take over and drown out other sounds (c.f. Section 3.3.1. Auditory Masking and Section 3.3.2. Visual Masking). Observations in this study indicate that the sound could be enhanced if the trees are surrounded by open land or planted as an avenue to allow the wind better access to the leaves, however no SPL readings could be taken to confirm this. In some gardens, like Funda-in, Chishaku-in, and Rikugi-en, it was apparent that trees planted on mounds had a strategic position to catch the wind. This was particularly evident in Funda-in and Chishaku-in, where the bamboo was planted on mounds:

A few moments of tense silence before a gust of wind starts to build up in the distance. At first, I can hear how the tall bamboo trees in the left back side of the garden start to move. They are strategically located on a high position which exposes them to more wind. The wind gust gradually approaches the garden, and I can now hear the rustle of the vegetation on the top of the hill, where a variety of species adds to the composition. Eventually, I can feel the wind passing the veranda and hear how the shoji doors behind me shake slightly as a response, before it quiets down again.

Chishaku-in; 21 May 2018 (Video S6a)

Bamboo has previously been acknowledged for its ability to invite sound into a garden, in China [84] as well as in Japan [44]. When describing the soundscape design in Rentaroh Taki’s Memorial garden in Taketa, Torigoe mentions how bamboo makes “sound when blowing in the wind, rustling its leaves and squeaking its stems” [44] (p. 112).

#### 3.3.5. Walking Materials

*Description:* Materials like gravel and wood are known to generate ample sonic responses when walked upon, while other materials like asphalt and stone are quieter. Furthermore, different materials can contribute with different connotations which have bearing on preference [86]. The sound of other people walking may be experienced as a pleasant atmosphere, or in some cases, as a warning [85].

*Gardens of particular interest:* Rentaroh Taki’s memorial garden (81), Nomura-ke samurai teien (70), Ryōan-ji (38), Shoren-in (47), Nijō-jō (34).

*Findings:* One of the most typical materials used to produce walking paths in Japanese gardens is no doubt stone. Stones are used in different ways: sometimes to create wide, cohesive and formal paths and sometimes as informal stepping stones carefully laid out in the ground, *tobi-ishi* or in water, *sawatari*. Stone paths do not produce so much sound today, though when they were originally conceived, traditional wooden shoes known as *geta* would have created a characteristic clonking sound [43,44]. This effect can still be experienced in Rentaroh Taki’s Memorial garden where such shoes are offered to visitors.

**Figure 12:**
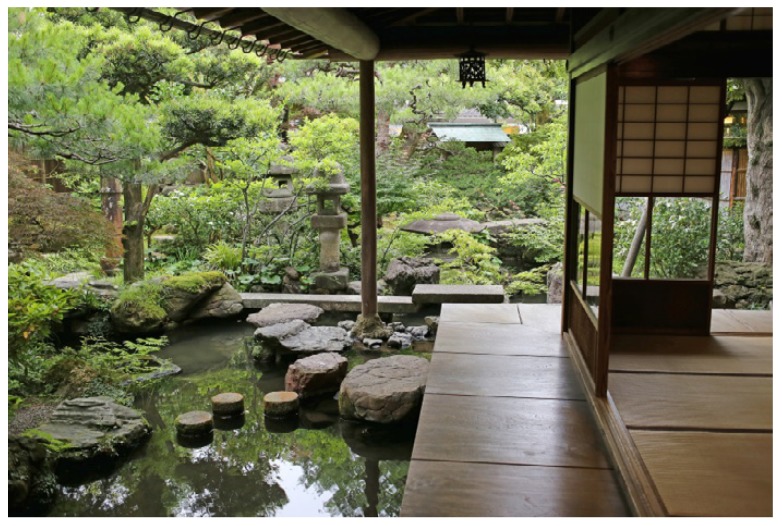
Nomura-ke Samurai teien in Kanazawa. View from the east overlooking the upper pond.

Another noteworthy feature is the extensive use of wooden floors. They are typically found in the verandas known as *engawa* (Figure 12). Some gardens, including the famous Ryōan-ji, are designed to be experienced from the *engawa*, where the thumping sound of wood becomes a characteristic *soundmark* [30]. It is quite common for the wooden verandas to extend into the garden as a system of roofed walkways that connect various buildings on the premises. Such walkways offer shade on hot days and shelter from precipitation, while the sound of thumping and squeaking floorboards constitute a potentially pleasant accompaniment, as the following example from Shoren-in illustrates:

The wooden walkways make for a nice stroll through the garden, as the roof provides shade in the sunshine. The floor is made from thick wooden floorboards; it has a nice, raw tactility to it. Each floorboard is slightly different in character; the thickness varies. Some floorboards make a heavy thumping sound when walked upon.

Shoren-in; 28 April 2018

The sound of the verandas’ wooden floors differs between gardens depending on the construction. One particularly noteworthy effect is the squeaking sound which has come to be called *uguisubari*, or nightingale floor, after the Japanese bush warbler, *uguisu* (*Horornis diphone)*, whose sound it supposedly resembles (Video S7, https://vimeo.com/311380259). Legend has it that the sound of the *uguisubari* was used to warn inhabitants of assassins and other unwelcome guests. This particular function may have played out its role today, yet there may be related applications in other contexts. For instance, in a study reporting on the role of soundscape in a rehabilitation garden, it was found that a patient who wanted to experience “social quietness” used the sound of a gravel path as a warning system from the potential approach of other patients in the garden [85]. Gravel paths can also be found in Japanese gardens, though the extensive use of stone and wood makes it less of a characteristic feature.

#### 3.3.6. Sound Sculptures

*Description:* A sound sculpture is a multisensory embellishment where sound plays an important part. There are many types of sound sculptures, and they can be driven by sources such as wind, water or electricity (speakers). The sound is usually accompanied by some sort of visual container that is typically designed to be a part of the expression.

*Gardens of particular interest:* Ōhashi-ke (35), Enkō-ji (9), Taizō-in (51), Giou-ji (14), Shisen-dō (45).

*Findings:* Two kinds of garden features that qualify as sound sculptures were studied: *suikinkutsu* and *sōzu (shishi-odoshi)*.

**Figure 13:**
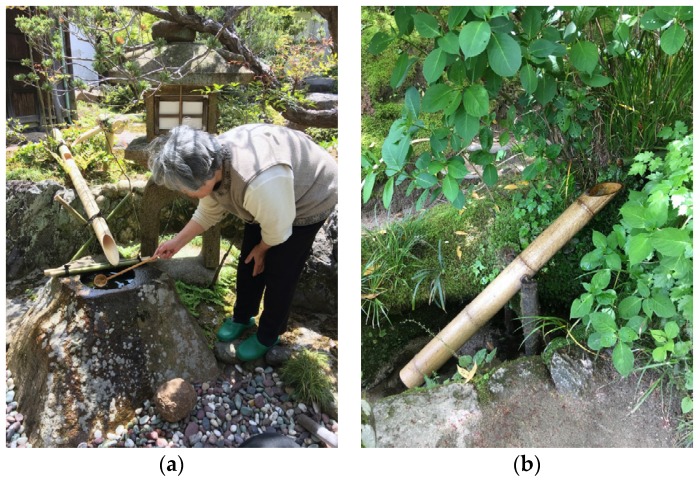
Two typical sound sculptures found in Japanese gardens. (**a**) A suikinkutsu in Ōhashi-ke, Kyoto (Video S8b,). (**b**) A *sōzu*, also known as *shishi-odoshi* in Shisen-dō, Kyoto (Video S9a).

*Suikinkutsu* is a simple but elegant design element that can be found in conjunction with some washing places, *tsukubai* (Figure 13a). The sound is created as excess water from the water basin, *chozubashi*, is led to drop into a hidden cavity below ground. The cavity essentially consists of a pot which is turned upside down and buried in the ground. The sound is generated at the bottom of the cavity where the drops break a water surface. The sound can be described as metallic and vibrant, fresh and melodic. The sound of *suikinkutsu* was experienced and documented in four gardens (Video S8a–e, https://vimeo.com/showcase/5683754). As a sound sculpture, *suikinkutsu* is unusual, as part of it is hidden beneath ground.

A *sōzu* is a contraption originally developed by farmers to keep deer, boars and other wild animals away from their fields (Figure 13b). It was subsequently incorporated as a design element in gardens [43]. It consists of a bamboo tube, which is opened up on one side to allow water to flow inside. The tube is mounted on a rack that works as a hinge so that the tube can move freely around the axes. The open end of the tube is located beneath a water supply. As the tube fills up, its center of gravity gradually shifts until it passes the hinge, causing the contraption to fall over, emptying its water and then making a distinct sound as it falls back to its resting position (Video S9a,b, https://vimeo.com/showcase/5683758). The *sōzu* is often called a *shishi-odoshi,* in which case a more overarching reference is inferred; both terms are generally translated as deer scarer.

#### 3.3.7. Biotope Design

*Description:* Biotopes and natural ecosystems can have positive effects on the soundscape, including the presence of song birds and other animals [87]. Song birds are generally attracted by basic features like access to water, shelter and food (most typically insects, seeds and berries) [88,89]. A general guideline is that birds prefer vegetation that is multi-layered, dense and variated.

*Gardens of particular interest:* Rurikoin (37), Shisen-dō (45), Sanbō-in (41), Shin’en (44) Rurikoin (37), Nanzen-in (32), Shisen-dō (45), Rentaroh Taki’s memorial garden (81).

*Findings:* Many of the studies of Japanese gardens were conducted during April and May, which provided ample opportunities to experience birds and other animals, particularly in gardens that bordered forests, such as Rurikoin, Shisen-dō and Sanbō-in, or gardens that included woodlands, such as Shin’en (Heian Jingu Shrine). Some species, like heron, seemed to enjoy sitting on stones laid out in ponds. It has previously been suggested that such stones were deliberately laid out in shallow water with the purpose of attracting birds [43]. Prerequisites for birds may also be increased by the use of vegetation, particularly fruit-bearing plants [44].

Spring is also the mating season for frogs, and the sometimes intense croaking could be experienced in pond gardens like Rurikoin, Nanzen-in and Shisen-dō. The effect was particularly breathtaking on May 15th 2018 in Nanzen-in, where the whole system of ponds seemed to be “illuminated” by croaking frogs, creating an intense spatial presence (Video S10, https://vimeo.com/270885125). Frogs may in fact have been actively placed in Nanzen-in when the garden was created in the late Kamakura period, as indicated by 15th century sources accessible to Ueyakato Landscape, that manages the garden today [90].

**Figure 14:**
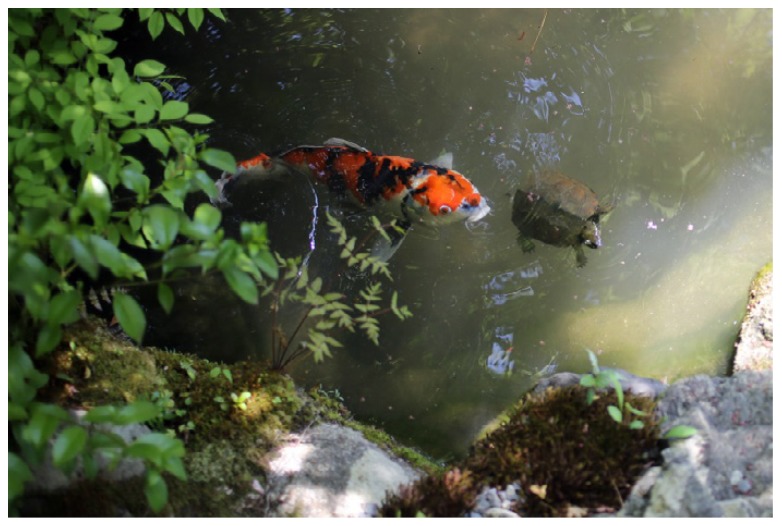
A turtle and a carp fish in Konchi-in temple garden, Kyoto.

Two other animals are commonly placed in Japanese gardens: turtles and carp fish (Figure 14). Almost all ponds encountered in the study had carp fish, while turtles were less common. In some gardens, for example Konchi-in, carp and turtles live side by side, creating spectacular sightings and the occasional splash. Both carp fish and turtles can make rather loud splashes, an effect that amuses visitors and possibly stimulates a heightened and active “listening” to the environment (as compared to a passive “hearing”) [91].

#### 3.3.8. Attract Activities

*Description:* This Soundscape Action acknowledges the relationship between landscape functions and sound by emphasising the fact that the introduction of new functions may generate activities that benefit the soundscape. A typical example of such a function is a café, the atmosphere of which can also be a quality for people passing by.

*Gardens of particular interest:* Chishaku-in (5), Daisen-in (7), Konchi-in (27), Nanzen-ji (33), Rurikoin (37), Tenryū-ji (52), Suizen-ji Joju-en (77).

*Findings:* Ticket sales, souvenir shops and cafés constitute some examples of recurring activities noted in the study. Religious activities could be experienced in gardens located in (or in the vicinity of) Buddhist temples and Shinto shrines. Even if secluded, the sound of religious activities would easily travel through the thin walls of the traditional buildings, making it customary to hear monks’ chanting or the distant ringing of a gong as a faint, mystical atmosphere. For the visitor, these atmospheric tints may constitute a reminder of the sacred context in which the gardens are located. The act of praying is ritualised in Shinto shrines as well as in Buddhist temples. In both cases, praying involves throwing a coin into an offering box, *saisen-bako*: an event which can create a rather loud sound if the box has resonating qualities.

A *temizuya* is a kind of water basin, where visitors go to purify themselves before a visit. The purification is carried out with the aid of a wooden ladle which is used to pour water on one’s hands and mouth. The *temizuya* generates a rather specific soundscape typically involving the murmur of people, the sound of water splashing on the ground and the distinct wooden clonks as the ladles are put down. The *temizuya* are typically associated with Shinto shrines though can also be found in Buddhist temples.

To sum up, the sound of activities signals the presence of other people and may be charged with symbolic meaning and atmospheric qualities. In design situations, such aspects should be given consideration in relation to the intended purpose. Social activities may be regarded as a quality in many contexts, yet may also be problematic in some situations. This is the case in gardens intended for recovery from stress-related illnesses, where seclusion from some forms of social activities is required [85,92].

#### 3.3.9. Resonance/Reflection

*Description:* Resonance/Reflection is concerned with how acoustically hard materials can be used to enhance wanted sound sources. For instance, if a concrete wall is strategically located behind a water feature, the sound of water can be enhanced by the reflections the wall creates.

*Gardens of particular interest:* Sanbō-in (41), Konchi-in (27), Shoren-in (47), Murin-an (31).

*Findings:* These kinds of acoustical effects were mainly found in and around water features, typically enforced by stones enhancing and directing the sound of water in gardens like Sanbō-in, Konchi-in and Shoren-in. The design intentions underlying these cavities could be several, and resonance may not have been the main one. Some of these cavities also worked to direct the sound, as the following notation from Konchi-in in Kyoto indicates:

The use of the grotto has other advantages as well. Not only is the sound amplified through the resonance it creates, but the grotto also seems to have a parabolic shape leading the sound across the pond towards the entrance.

Konchi-in; 21 April 2018

This way of directing sound opens up for a wide range of creative possibilities and experiential effects. In the above example, the sound of the waterfall is best experienced from the opposite (northern) side of the pond, yet because it cannot be seen from that point, an anticipation is created that encourages further exploration of the garden in search for the source. It has previously been described how the sound of hidden water features can be used to create a “spatial appeal” that invites a visitor to go from a “here” to search for a “there” [41]. Such kind of explorations seem to be related to the notion of “soft fascination” as described by Kaplan and Kaplan, as part of their attention restoration theory (c.f. [48,85,93]).

## 4. Discussion

### 4.1. Soundscape Actions as a Design Tool

The Soundscape Actions presented in the study constitute a collection of examples, illustrating how landscape architects and other professionals could think about soundscape design in their work [27,36]. Covering a total of 23 Soundscape Actions divided in three categories, it is an extensive framework. This emerged as beneficial in the study, as it provided a variety of entry points from which to understand the soundscapes of Japanese gardens. In this way, aspects that would not necessarily have been noted otherwise could be highlighted.

In the study, 19 of the 23 Soundscape Actions were applicable. The other four (Embrace unwanted sounds, Abolish certain functions, Low noise screens and Atmospheric design) were excluded because no concrete examples were encountered. This is partly owing to the particular character of Japanese gardens, where certain expressions are not likely to be found, such as urban vibrancy (Embrace unwanted sounds) or artificial speaker installations (Atmospheric design). In future developments of the tool, it might be possible to take this into consideration by designing a system in which Soundscape Actions are tagged based on their appropriate application (for instance urban vibrancy, traffic planning and parks/gardens). The other two excluded actions seem relevant for Japanese gardens, yet could not be registered in the study. “Abolish certain functions” (such as roads, factories or air conditioners) should be a fruitful strategy to enhance garden experiences, however no clear examples were identified (in part, owing to language difficulties). “Low noise screens” is a novel solution for traffic noise reduction and only a limited number of practical applications are known worldwide.

Some sonic experiences that were noted in the study did not correspond directly to any of the 23 Soundscape Actions. These experiences could generally be described to be of a phenomenological nature, including effects related to movement, orientation, behaviour and subjective contemplation. This discrepancy may partly be regarded as a consequence of the autoethnographic approach, in which such experiences should be expected to be brought forward; it might also be taken to indicate specificities associated with the context being studied, i.e., Japanese gardens. The encountered effects were referred to in the study in conjunction with related Soundscape Actions (for example subtle water features in Section 3.3.3 and interactive walking materials in Section 3.3.5). There seems to be scope to formulate new Soundscape Actions. This potential will be further examined in upcoming publications.

### 4.2. Implementation and Usage

The Soundscape Actions constitute a set of strategies with the potential to improve experiential qualities in gardens and other urban (green) spaces. However, it should be noted that, because each situation is unique, the effect of Soundscape Actions will vary, and all of them will not necessarily lead to an improvement every time. One of the challenges of soundscape design is to understand complex relationships and the degree to which various factors effect a situation. The tool brings together a collection of central aspects as a framework that can be used to build an experience base. In future work, this base could be further developed by applying the tool in other contexts and with other participants. The material thus generated could be collected and presented online for accessibility. A digital platform suitable for this purpose already exists (https://soundscapedesign.info). Practitioners should be involved in future developments so as to safeguard compatibility with established working patterns. One way forward could be to evaluate the tool in design workshops and/or use it to build an intervention (which could subsequently be evaluated). Future work could also explore the possibility of integrating the tool with computer aided calculations, known as auralisation. This would potentially allow for real time comparisons between Soundscape Actions in different scenarios.

The study focused on tranquil soundscapes in Japanese gardens, taking into consideration the potential for negative as well as positive health effects [24,25]. The study did not assess health effects per se, but rather used existing research to envisage the effects. As discussed above, there is a strong body of research showing that exposure to noise increases stress and leads to a number of severe negative health effects [24]. This should be taken in account when designing outdoor spaces. Problems with noise have primarily been addressed by engineers and acousticians, yet much can also be achieved by urban planners and designers [27,28,32,94,95]. Increased collaboration across disciplines could further enhance prerequisites for health promoting environments. In terms of positive health effects, there is an increasing number of studies indicating that exposure to nature sounds have beneficial effects on health [25]. This should also be given consideration in design situations so that such sounds are afforded the best possible prerequisites. The present study provided several examples of how this could be accomplished.

## 5. Conclusions

This study has focused on the Japanese garden tradition in order to gain insights on the design tool Soundscape Actions, and how it can be used to improve soundscapes in gardens and other green spaces. Field studies were conducted in 88 Japanese gardens, and notated experiences from (in total) 136 visits to those gardens were used as empirical material. Most of the analysis and presentation centred on gardens located in Kyoto (n = 54), as this allowed for comparisons within the city.

The research was designed using an autoethnographic approach [45]. This made it possible to obtain detailed understandings on the intersection between landscape architecture and soundscape research. Autoethnography emphasises subjectivity and therefore allows for embodied experiences to be brought forward. This enables the researcher to go into detail and consider the interplay between senses and various site-specific factors. It should be pointed out that the subjective nature of the method could limit the applicability of the findings in other contexts. To increase generalisability, supportive data was collected in situ, including SPL readings, images, videos and field recordings. This material has been made available in the paper, and should mitigate interpretation. Where applicable, findings were compared and discussed in relation to previous research. Yet, caution should be taken when interpreting the results, and future studies involving more participants are recommended to validate the findings.

Sound constitutes one of several aspects of the environmental experience, and it should be stressed that the interplay between the senses is of importance, even when focusing on a particular sense [85]. The study highlights the role of such relationships when discussing masking and other complex mechanisms. Masking is based on the assumption that sounds experienced as positive can be used to reduce the annoyance caused by traffic and other (unwanted) technological sources [72,74,82]. The benefits of masking have been debated, partly because the underlying mechanisms are not fully understood. This study indicated that spatial context and multisensory impressions play an important role, and that such factors should be given increased consideration in future research. The subjective experience of individuals seems to be essential if these effects are to be uncovered further. Subjectivity is emphasised in the ISO definition of soundscape [34], yet has been given surprisingly little attention in research thus far.

Much effort has been invested to analyse and uncover the knowledge existing in the Japanese garden tradition, though most previous publications have tended to prioritise visual aspects. The present study illustrates how Japanese gardens can also be informative regarding sound. The findings presented here follow the Soundscape Action design tool and cover a wide array of perspectives. The study illustrates multiple strategies to avoid noise, such as the use of remote locations, garden walls and absorbent moss. Reduction of noise allows for other and more delicate experiences to come forward, like sounds of nature. Sounds of nature constitute an essential element in Japanese gardens and they can be articulated and enhanced in design solutions. This is highlighted in the study through the way that rustling trees are used, how animals are attracted and how the sound of water is orchestrated to produce loud masking sounds. Taken together, the soundscapes of Japanese gardens are surprisingly tranquil considering the modern and densified cities that often surround them.

## Figures and Tables

**Table 1:** Equipment and processes used to notate sonic experiences in the gardens.

Type of Data	Description
Field notes	The most central piece of equipment was a small (analogue) notebook that was used to list garden encounters, focusing on soundscapes and sonic events in relation to landscape architecture. Notes were continuously transcribed digitally in a Microsoft Word document.
Images and video	To capture photos, a Canon EOS 6D DSLR camera was used together with a 35 mm f/2 lens and (during autumn 2018) a Canon EF 28–105 mm f/3.5–4.5 USM lens. In addition, the built-in camera of an IPhone SE was used to capture panorama and HDR images and video. Video was recorded at HD (1080 p, 30 fps).
Field recordings	A Zoom H2n was used to record sound, and in most cases the built-in microphones were used in the XY setting together with a thick wind screen. In cases where a wider spatial effect was deemed necessary, a pair of external Roland CS-10 EM binaural microphones was used. The quality was set to 24 bits at 44 kHz at all times.
Sound Pressure Levels (SPL)	SPLs were measured with an IPhone SE (internal microphone) together with the application NIOSH SLM (Version 1.0.6.24) to obtain approximated instantaneous readings in dBA ^1^. Instantaneous readings were taken at approximately 1.5 metres above the ground for a few seconds; care was taken to protect the device from direct wind exposure and hand noise. In cases where a particular source was evaluated, the microphone was directed towards that source.

^1^ The setting had been tested prior to the study in an urban environment sheltered from wind (SPL varying between 50–65 dBA). A Norsonic 140 (Class 1) SPL meter was used as control, and the manual setting in NIOSH SLM was calibrated to −2.1 dBA. After the study, the setting was tested again, this time by playing back different types of noises indoors and using a Brüel & Kjaer 2270 (Class 1) SPL meter as control. This test indicated that the setting had been satisfactory for the situations observed in the study (35–65 dBA), where the discrepancy did not exceed ±1.5 dBA. With this being said, it should be noted that SPL readings taken with smartphone applications do not comply with international standards. Studies on smartphone applications for SPL measurements have shown that, even though some of them may be suitable to measure occupational noise [59], the performance varies and limitations in accuracy can be problematic in the wrong context [60]. The SPL readings should be interpreted with this in mind.

## Supplementary Materials

The following are available online at https://www.mdpi.com/1660-4601/16/23/4648/s1, Video S1: Compensation/Variation in Ōhashi-ke; Video S2: Spring in Sanbō-in; Video S3: Screening and Masking in Murin-an; Video S4: Muffled Waterfall in Rikgui-en; Video S5: Enhanced Water Stream in Shin’en; Video S6a: Water and Wind in Chishaku-in; Video S6b: Subtle Water Drops in Ginkaku-ji; Video S7: Nightingale Floors; Video S8a: Suikinkutsu in Ōhashi-ke, 1/2; Video S8b: Suikinkutsu in Ōhashi-ke, 2/2; Video S8c: Suikinkutsu in Enkō-ji; Video S8d: Suikinkutsu in Taizō-in; Video S8e: Suikinkutsu in Giou-ji; Video S9a: Shishi-odoshi in Shisen-dō; Video S9b: Shishi-odoshi in Taizō-in; Video S10: Frogs in Nanzen-in.

## Appendix A

This appendix of Japanese gardens provides detailed information about all study visits that were carried out (Table A1).

**Table A1:** An overview indicating the numbers, names and locations of the studied gardens, as well as number of visits, dates for visits and total time spent in each garden.

Nr	Garden’s Name	Location	Number of Visits	Date/s for Visits	Total Time Spent (min)
1	Hōsen-in	Ohara, Kyoto	1	10 April 2018	110
2	Sanzen-in	Ohara, Kyoto	1	10 April 2018	40
3	Shorin-in	Ohara, Kyoto	1	10 April 2018	40
4	Byōdō-in	Uji, Kyoto	1	12 December 2018	60
5	Chishaku-in	Kyoto	3	21 April 2018, 21 May 2018, 29 November 2018	240
6	Daikaku-ji	Kyoto	1	02 December 2018	70
7	Daisen-in	Kyoto	2	06 April 2018, 06 December 2018	60
8	Eikan-dō	Kyoto	2	18 April 2018, 29 November 2018	120
9	Enkō-ji	Kyoto	3	08 April 2018, 12 May 2018, 27 November 2018	315
10	Entsū-ji	Kyoto	1	19 April 2018	40
11	Fumon-in	Kyoto	2	24 April 2015, 30 April 2018	30
12	Funda-in	Kyoto	1	30 April 2018	100
13	Ginkaku-ji	Kyoto	3	23 April 2015, 04 April 2018, 20 December 2018	130
14	Giou-ji	Kyoto	1	02 December 2018	60
15	Gonaitei (Imperial palace)	Kyoto	1	01 December 2018	45
16	Goten (Ninna-ji)	Kyoto	1	02 December 2018	75
17	Hōnen-in	Kyoto	1	12 April 2018	30
18	Horai-no-niwa (Matsuno’o Taisha)	Kyoto	1	13 December 2018	40
19	Jōjuin	Kyoto	1	29 November 2018	45
20	Jōkō-no-niwa (Matsuno’o Taisha)	Kyoto	1	13 December 2018	40
21	Katsura Rikyū	Kyoto	2	24 April 2015, 04 April 2018	120
22	Kegon-ji	Kyoto	1	15 April 2018	30
23	Kennin-ji	Kyoto	1	16 April 2018	80
24	Kinkaku-ji	Kyoto	2	22 April 2015, 01 December 2018	60
25	Koetsu-ji	Kyoto	1	01 December 2018	20
26	Kōmyō-in	Kyoto	1	13 April 2018	30
27	Konchi-in	Kyoto	2	21 April 2018, 14 May 2018	150
28	Konpuku-ji	Kyoto	1	26 April 2018	30
29	Kyokusui-no-niwa (Matsuno’o Taisha)	Kyoto	1	13 December 2018	40
30	Manshu-in	Kyoto	1	27 November 2018	60
31	Murin-an	Kyoto	7	23 April 2015, 25 April 2015, 06 May 2018, 14 May 2018, 20 May 2018, 20 November 2018, 08 December 2018	365
32	Nanzen-in	Kyoto	2	18 April 2018, 14 May 2018	60
33	Nanzen-ji	Kyoto	2	23 April 2015, 05 April 2018	60
34	Nijō-jō	Kyoto	1	07 April 2018	80
35	Ōhashi-ke	Kyoto	1	13 April 2018	50
36	Oikeniwa (Imperial palace)	Kyoto	1	01 December 2018	20
37	Rurikoin	Kyoto	2	19 April 2018, 19 November 2018	270
38	Ryōan-ji	Kyoto	3	22 April 2015, 06 April 2018, 02 December 2018	160
39	Ryosoku-in	Kyoto	1	30 May 2018	20
40	Saihoji (Kokedera)	Kyoto	2	15 April 2018, 18 November 2018	270
41	Sanbō-in	Kyoto	1	23 April 2018	60
42	Sanjūsangendō	Kyoto	1	26 November 2018	15
43	Sentō Imperial Palace	Kyoto	2	18 April 2018, 05 December 2018	120
44	Shin’en (Heian Jingu)	Kyoto	2	09 April 2018, 05 May 2018	180
45	Shisen-dō	Kyoto	5	06 April 2018, 10 April 2018, 26 April 2018, 15 May 2018, 27 November 2018	240
46	Shōinan	Kyoto	1	23 April 2015	15
47	Shoren-in	Kyoto	2	23 April 2015, 28 April 2018	285
48	Shōsei-en	Kyoto	3	29 April 2018, 21 May 2018, 26 November 2018	210
49	Shūgaku-in Rikyū	Kyoto	2	19 April 2018, 06 December 2018	180
50	Shūsui-tei	Kyoto	2	16 March 2018, 05 December 2018	30
51	Taizō-in	Kyoto	1	18 December 2018	90
52	Tenryū-ji	Kyoto	2	20 April 2018, 18 November 2018	95
53	Tōfuku-ji	Kyoto	3	24 April 2015, 07 April 2018, 20 November 2018	145
54	Zuihō-in	Kyoto	1	06 December 2018	60
55	Denbo-in Teien	Tokyo	1	14 March 2015	15
56	Hama-rikyu	Tokyo	1	20 October 2018	60
57	Kakuuntei	Tokyo	1	31 August 2018	30
58	Kiyosumi Teien	Tokyo	1	19 January 2019	45
59	Koishikawa-Kōrakuen	Tokyo	2	02 August 2015, 03 June 2018	80
60	Kyū-Furukawa Teien	Tokyo	1	19 August 2018	30
61	Kyu-Shiba-rikyu	Tokyo	1	20 October 2018	85
62	Mukōjima-Hyakkaen	Tokyo	1	19 January 2019	15
63	Nezu museum garden	Tokyo	1	08 September 2018	60
64	Rikugi-en	Tokyo	5	02 August 2015, 31 March 2018, 19 August 2018, 19 October 2018, 20 January 2019	320
65	Shinjuku Gyoen	Tokyo	2	09 April 2015, 30 March 2018	150
66	Gyokusen-en teien	Kanazawa	1	14 June 2018	40
67	Gyokusen-in-maru	Kanazawa	1	14 June 2018	10
68	Kenroku-en	Kanazawa	2	14 June 2018, 18 June 2018	120
69	Kurando Terashima teien	Kanazawa	1	18 June 2018	40
70	Nomura-ke Samurai teien	Kanazawa	2	14 June 2018, 15 June 2018	80
71	Oyama shrine pond garden	Kanazawa	1	14 June 2018	10
72	Seisonkaku	Kanazawa	1	14 June 2018	40
73	Shofukaku	Kanazawa	1	15 June 2018	40
74	Takada family home garden	Kanazawa	1	15 June 2018	10
75	Tsujike teien	Kanazawa	1	14 June 2018	30
76	Kōrakuen	Okayama	1	24 April 2018	60
77	Suizen-ji Joju-en	Kumamoto	1	21 February 2019	90
78	Kusano Honke	Hita	1	19 February 2019	15
79	Adachi Museum Garden	Matsue	1	22 February 2019	240
80	Ritsurin Kōen	Takamatsu	1	24 February 2019	170
81	Rentaroh Taki’s Memorial garden	Taketa	2	20 February 2019, 21 February 2019	70
82	Isui-en	Nara	2	26 August 2015, 21 December 2018	100
83	Yoshiki-en	Nara	1	21 December 2018	50
84	Keitakuen	Osaka	1	15 December 2018	60
85	Takayama Jin’ya	Takayama	1	16 June 2018	30
86	Shukkeien	Hiroshima	1	15 July 2015	20
87	Shiki-naen	Naha	1	24 June 2018	100
88	Shuri-jo	Naha	1	24 June 2018	45
	**Total: 88 gardens**		**Total: 136 visits**		**Total: 7750 min (= 129 h)**
