# Supplementary material for: Listening to Japanese Gardens: An Autoethnographic Study on the Soundscape Action Design Tool
Source: Int J Environ Res Public Health. 2019 Nov 22;16(23):4648. doi: 10.3390/ijerph16234648 (PMC6926712; doi:10.3390/ijerph16234648)
Supplement: Supplementary file 1 [file ijerph-16-04648-s001.pdf]

Supplementary Material: List of Video files

Supplementary Material to the research paper entitled: "Listening to Japanese Gardens: An Autoethnographic Study on the Soundscape Action Design Tool". The following list comprises an overview of the audio-visual material referenced in the paper.

Video S1. Compensation/Variation in Ōhashi-ke.

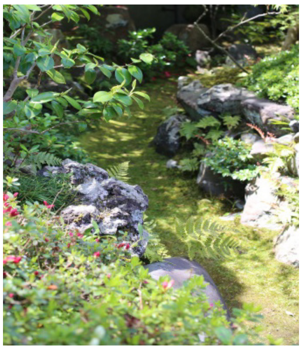
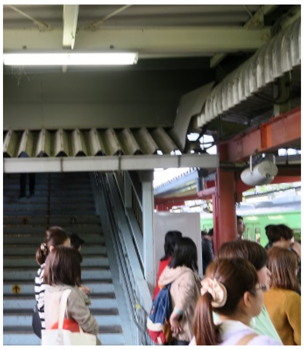

A juxtaposition of two soundscapes: the garden Ōhashi-ke and the busy Tofukuji station, both in the southeastern part of Kyoto. The video presents an opportunity to evaluate how the contrast between these soundscapes influences the experience.

Link: https://vimeo.com/350108144

Video S2. Spring in Sanbō-in.

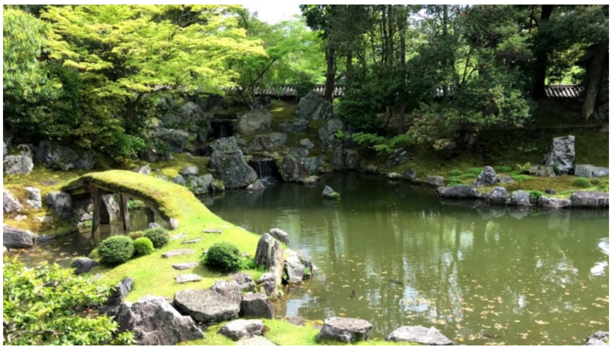

The soundscape of Sanbō-in garden in Kyoto, as experienced on a spring day in 2018. The soundscape includes a waterfall, birds, frogs, garden visitors and manual garden maintenance. The garden is located next to a woodland.

Link: https://vimeo.com/312665806

Video S3. Screening and Masking in Murin-an.

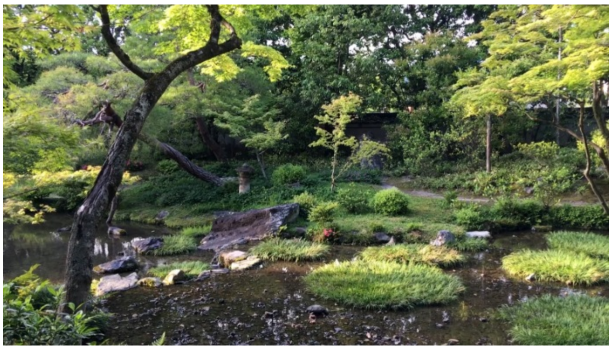

The eastern part of Murin-an garden, delimited by wall and vegetation. Traffic noise from a busy road that passes outside is reduced by the garden wall’s screening effect, as well as the masking sound of a waterfall. Binaural recording; use headphones to reproduce spatiality.

Link: https://vimeo.com/311434655

Video S4. Muffled Waterfall in Rikugi-en.

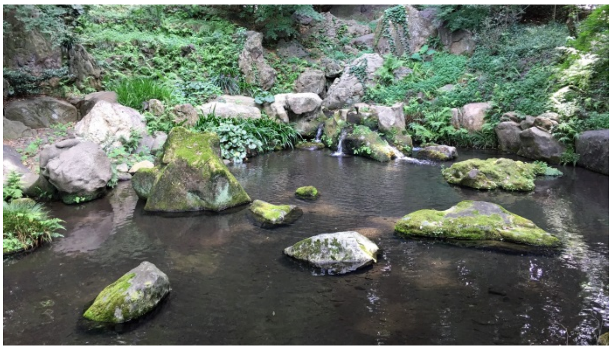

A small waterfall in Rikugi-en garden, Tokyo, as experienced from two different locations. In one of the locations (inside the tea house), the sound of the fall is muffled, arguably making it seem further away. Binaural recording; use headphones to reproduce spatiality.

Link: https://vimeo.com/312519101

Video S5. Enhanced Water Stream in Shin'en.

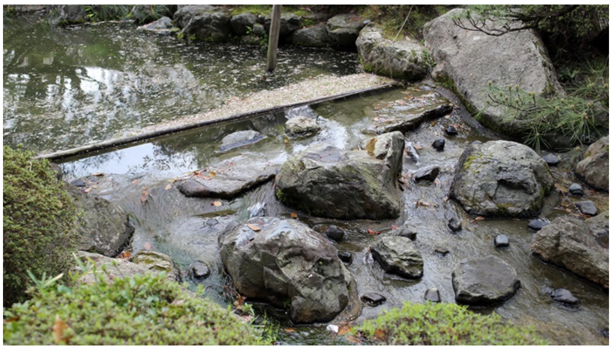

A stream in Shin'en garden (Heian Jingu Shrine, Kyoto), where rocks are strategically laid out to enhance the sound of water.

Link: https://vimeo.com/311170428

Video S6a–b: Subtle Water Features

Two examples of subtle sounds from water features.

Video S6a. Water and Wind in Chishaku-in.

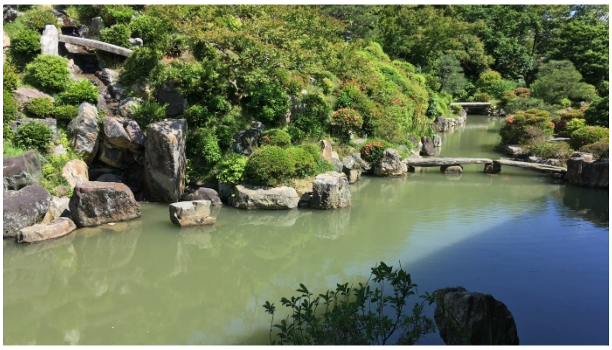

The soundscape of Chishaku-in garden, as experienced on a windy day in May 2018. The clip focuses on a trickling water feature as the wind plays in bamboo trees located on a hill in the far back of the garden.

Link: https://vimeo.com/311182675

Video S6b. Subtle Water Drops in Ginkaku-ji.

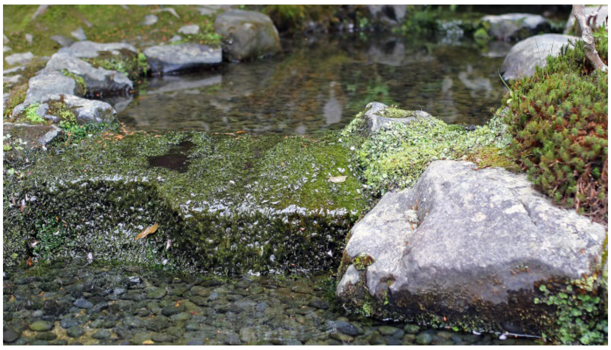

A stream in Ginkaku-ji with slow water flow and stones covered in moss. A barely audible sound is created as drops of water seep down to the next level.

Link: https://vimeo.com/311079936

Video S7. Nightingale Floors.

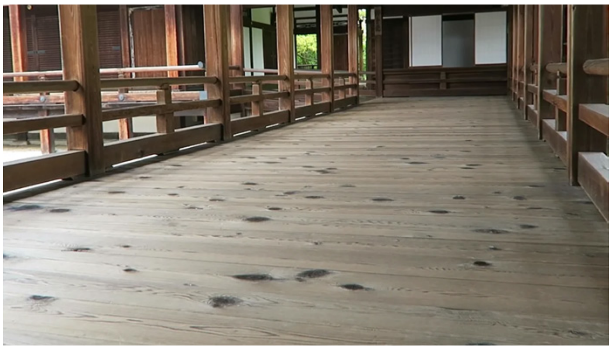

Documentation of nightingale floors, *uguisubari*, in Nijo-jo castle and Tōfuku-ji temple (both in Kyoto). The effect gets its name from the Japanese bush warbler, *uguisu*, the song of which is included in the clip for reference.

Link: https://vimeo.com/311380259

Videos S8a–e: Suikinkutsu

A collection of five *suikinkutsu* in four gardens.

Video S8a. Suikinkutsu in Ōhashi-ke, 1/2.

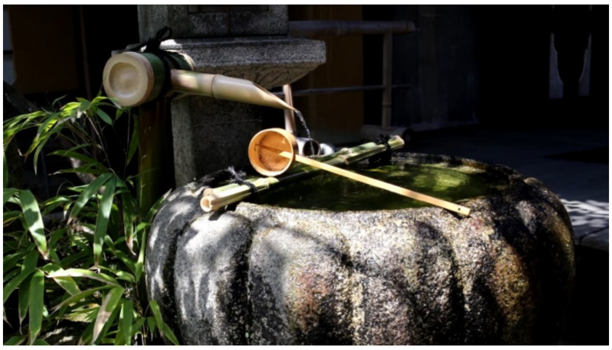

Ōhashi-ke was designed by the famous garden designer Ogawa Jihei in 1913. The garden's two *suinkutsu* are known as the oldest in Kyoto. This video documents the first one.

Link: https://vimeo.com/311192651

Video S8b. Suikinkutsu in Ōhashi-ke, 2/2.

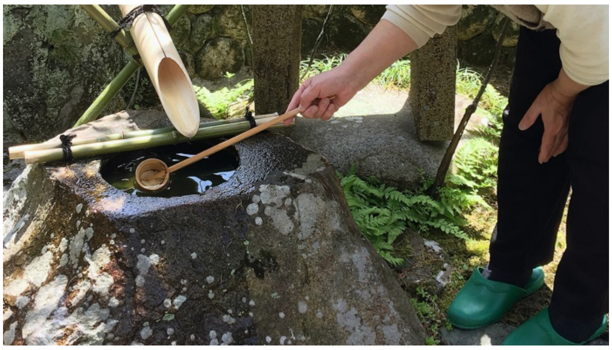

A demonstration of the second of two old *suikinkutsu* in Ōhashi-ke, Kyoto.

Link: https://vimeo.com/311170222

Video S8c. Suikinkutsu in Enkō-ji.

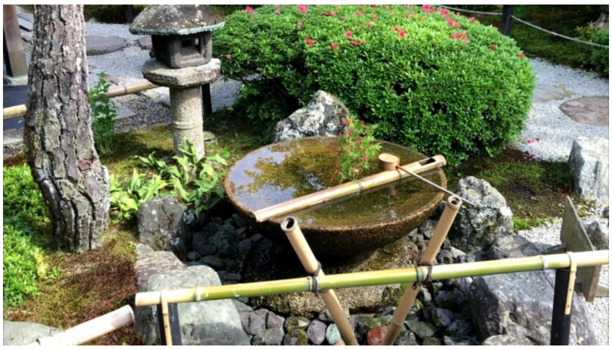

Enkō-ji temple garden lies in the northeastern part of Kyoto where it borders to forested mountains. The *suikinkutsu* is located close to the garden entrance.

Link: https://vimeo.com/311192441

Video S8d. Suikinkutsu in Taizō-in.

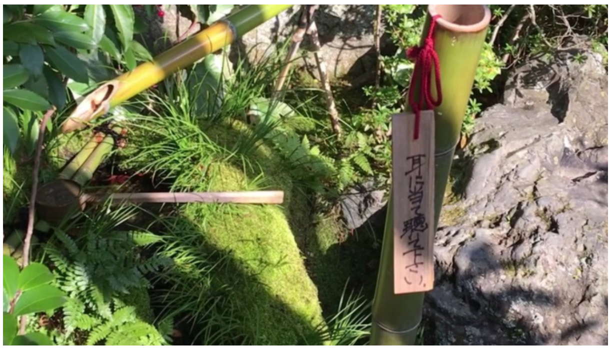

In the northwestern part of Kyoto lies Taizō-in garden. In addition to the *suikinkutsu* recorded here, the garden also has a deer scarer (Video 9b).

Link: https://vimeo.com/311178001

Video S8e. Suikinkutsu in Giou-ji.

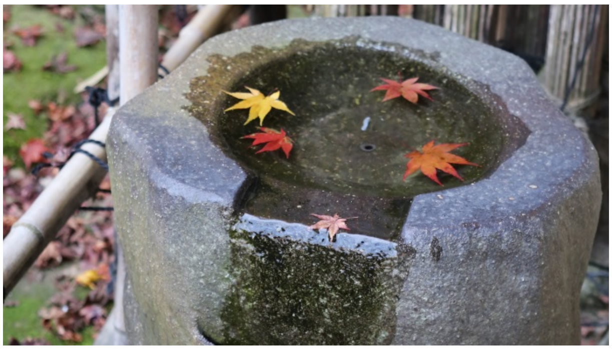

A stand-alone *suikinkutsu* in Giou-ji, northwestern Kyoto.

Link: https://vimeo.com/311177932

Videos S9a–b: Shishi-odoshi

Two examples of deer scarers, *sōzu* a.k.a. *shishi-odoshi.*

Video S9a. Shishi-odoshi in Shisen-dō.

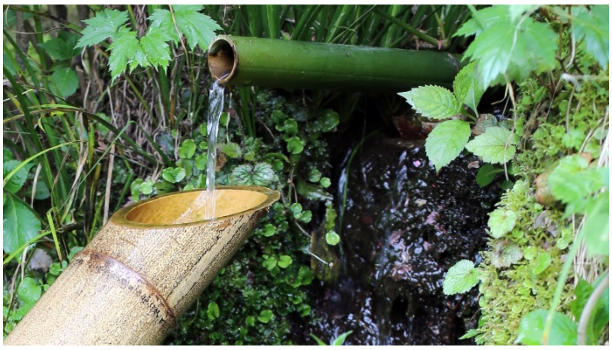

The *shishi-odoshi* was originally used to scare wild animals. Shisen-dō is known as one of the first gardens to embrace the contraption as part of the garden aesthetics. In this recording from April 2018, it takes about 30 seconds for the device to complete a cycle.

Link: https://vimeo.com/311374464

Video S9b. Shishi-odoshi in Taizō-in.

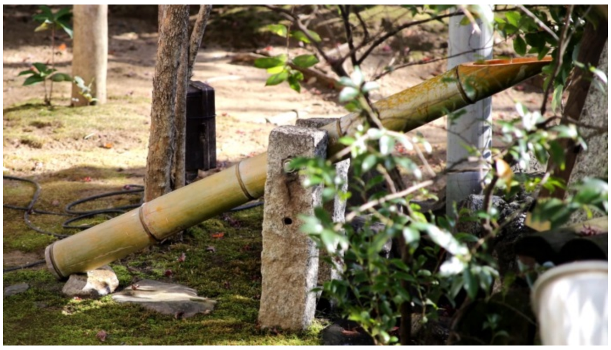

This large *shishi-odoshi* in Taizō-in, Kyoto, has an interval between strikes of about 4 minutes.

Link: https://vimeo.com/311374391

Video S10. Frogs in Nanzen-in.

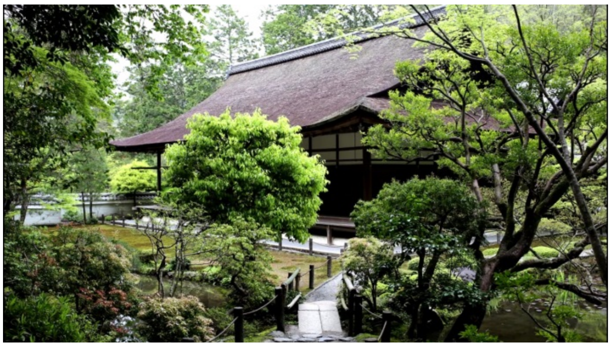

Frogs croaking intensely in Nanzen-in temple garden, Kyoto. Binaural recording; use headphones to reproduce spatiality.

Link: https://vimeo.com/270885125
